# The diagnostic value of homocysteine for the occurrence and acute progression of chronic obstructive pulmonary disease

**DOI:** 10.1186/s12890-020-01265-w

**Published:** 2020-09-07

**Authors:** Bing Wei, Tian Tian, Yugeng Liu, Chunsheng Li

**Affiliations:** 1 Department of Emergency Medicine, Beijing Chao-Yang Hospital Jingxi Branch, Capital Medical University, No.5 Jingyuan Road, Shijingshan, Beijing, 100043 China; 2 Department of Emergency Medicine, Beijing Chao-Yang Hospital, Capital Medical University, No.8 Gongti South Road, Chaoyang District, Beijing, 100020 China

**Keywords:** COPD, Homocysteine, Acute progression, Occurrence

## Abstract

**Background:**

This study aimed to evaluate whether the Homocysteine (Hcy) level was elevated in chronic obstructive pulmonary disease (COPD) patients and its correlation with the occurrence and acute progression of COPD.

**Methods:**

From November 2014 to November 2015, COPD patients were enrolled from Beijing Chao-yang Hospital, and the the biological and clinical data were collected. These patients were tested in the non-acute exacerbation period and the acute exacerbation period, so they were defined as AECOPD group and Non-AECOPD group. Besides, 50 healthy subjects were recruited and defined as control group. Total plasma Hcy levels (antibodies-online, USA) were determined by enzyme-linked immunosorbent assay. Correlation analysis was used to analyze the correlation between serum Hcy level and ventilatory function. Using ROC curve, the diagnostic value of Hcy for the occurrence and acute progression of COPD was explored.

**Results:**

In this study, we found that Hcy levels in the Non-AECOPD group or the AECOPD group were significantly higher than those in the control group (*P* < 0.001). Meanwhile, compared with the Non-AECOPD group, the Hcy level in the AECOPD group was significantly higher (*P* < 0.001). In addition, according to the classification of GOLD grade, there was significant difference in the Hcy level among different GOLD grade groups (*P* < 0.001). The correlation analysis showed that in the AECOPD group and the Non-AECOPD group, Hcy levels presented a negative correlation with FEV1(*r* < 0). Meanwhile, FEV1% was also negatively correlated with Hcy level (*r* < 0). ROC curve analysis showed that when the cutoff value was set to 10.8 μg/ml, the specificity, sensitivity and AUC were the best, which were 0.980, 0.800, and 0.945, respectively. Besides, our results showed that when the cutoff value was set to 14.0 μg / ml, the specificity, sensitivity and AUC were the best, which were 0.846, 0.680, and 0.802, respectively. In addition, compared with the prediction of acute progression of COPD, when Hcy level predicted the occurrence of COPD, its specificity (0.980 vs. 0.846, *P* < 0.001) and sensitivity (0.800 vs. 0.680, *P* < 0.001) were significantly higher.

**Conclusion:**

Hcy level is positively correlated with the severity of COPD patients, which has predictive value for the occurrence of COPD and acute progression.

## Background

Chronic obstructive pulmonary disease (COPD) is a chronic inflammation disease of the respiratory system, which is characterized by persistent airflow obstruction, accelerated loss of lung function and irreversible pulmonary damage. According to the estimation from WHO, 5% of deaths can be attributed to COPD in the world. Patients with COPD always show a sudden deterioration in lung function, termed acute exacerbation COPD (AECOPD), which is characterized by various rapidly worsening respiratory symptoms that can have a negative effect on quality of life and an increased risk of mortality [1].

Homocysteine (Hcy) is a sulfur-containing amino acid, which is an intermediate metabolite produced in the process of decomposition of methionine [2]. hyperhomocysteinemia is caused by defects in the enzyme system associated with Hcy synthesis and metabolism and nutrient deficiencies, such as folic acid [3] and Vitamin B12 (VITB12) [4]. The elevation of Hcy level can lead to cerebral infarction, coronary heart disease, hypertension, and other cardiovascular and cerebrovascular diseases [5]. It has been reported that increased expression of Hcy can accelerate the development of the disease by causing damage to vascular endothelial cells [6]. More and more evidences have shown that elevated plasma Hcy levels may also contribute to poor prognosis of COPD patients [7–9]. In addition, studies have indicated that other methods of reducing total Hcy level may yield different results on serum inflammatory markers [3]. However, our previous findings suggested that an imbalance of circulating CD4^+^ T cell subsets correlates with AECOPD, and that a shift of Th1/Th2 and IL-17/IgE ratios may be caused by increased Th2 cell production [1]. Therefore, Hcy may be involved in the pathogenesis of COPD patients through regulating inflammatory factor in vivo.

Herein, we investigated whether the Hcy level was elevated in COPD patients and its correlation with the occurrence and acute progression of COPD, which aimed to explore its diagnostic value and provide clinical references for the occurrence and acute progression of COPD.

## Methods

### Patients

From November 2014 to November 2015, the biological and clinical data were obtained from COPD patients, who were enrolled from Beijing Chao-yang Hospital. In this retrospective study, these patients were tested in the non-acute exacerbation period and the acute exacerbation period, so the data of different stages were defined as AECOPD group and Non-AECOPD group. In addition, 50 healthy subjects with normal pulmonary function were recruited, who were defined as control group. The written informed consent was obtained from all subjects, and the protocol was approved by the human research ethics committee of Beijing Chao-Yang Hospital (reference number: 2014-KE-124).

Each patient was given a COPD assessment test (CAT) [10] and a questionnaire (supplementary file 1) with eight questions concerning the presence of coughing, sputum, chest distress, asthma, and activity limitations caused by COPD at home and outdoors. For each question, a score of 0–5 was selected by the patients. The total score (0–10: slight effect; 11–20: median effect; 21–30: severe effect; 31–40: extreme severe effect) was then calculated to determine the effect of COPD on the daily activity level. A modified UK Medical Research Council (mMRC) eval- uation was used to assess dyspnea levels in COPD patients. An mMRC score of 2 was considered to be an indication of severe dyspnea.

#### Inclusion criteria

All patients were evaluated according to the diagnostic criteria of the Global Initiative for Chronic Obstructive Lung Disease (2015) as previously study [1]. 1) COPD: patients with difficulty in breathing, with a cough, sputum, and pulmonary function testing showing a post- bronchodilator forced expiratory volume in 1 s (FEV1)/forced vital capacity ratio < 70% and FEV1 < 80%; 2) AECOPD: a sudden worsening of COPD symptoms (shortness of breath, quantity and color of phlegm) that typically last for several days.

#### Exclusion criteria

(1) using oral steroids; (2) with apparent infections (urinary tract infections or pneumonia) or signs of sepsis; (3) had chronic diseases such as diabetes, renal failure; (6) with cancer or any other respiratory diseases.

### Sample collection and enzyme-linked immunosorbent assay

3 ml peripheral venous blood samples were collected from all study subjects admitted to hospital within 24 h. The serum was centrifuged at 1800 rpm for 20 min and stored at − 80 °C. Total plasma Hcy levels (antibodies-online, USA) were determined by enzyme-linked immunosorbent assay according to the manufacturers’ recommendations.

### Correlation analysis and diagnostic value test

Spearman correlation analysis was used to analyze the correlation between serum Hcy level and ventilatory function, and *r* value was calculated. r > 0 shows a positive correlation, while *r* < 0 shows a negative correlation.

Using ROC curve, the difference in Hcy level between the AECOPD group and the Non-AECOPD group, as well as the difference between the Non-AECOPD group and control group, were analyzed to explore the diagnostic value of Hcy for the occurrence and acute progression of COPD. Besides, its sensitivity, specificity, positive predictive rate (PPV), negative predictive rate (NPV), Youden’s index and Area under the curve (AUC) were calculated for further analysis.

### Statistical analysis

SAS9.3 software was used to analyze the data. Normally distributed measurement data were expressed as mean ± standard deviation (SD), while non-normally distributed measurement data were expressed as median (interquartile range), and the comparisons were examined by Student’s t-test and Wilcoxon two sample test. The difference between AECOPD group and Non-AECOPD group was analyzed by Wilcoxon signed rank test. One-way ANOVA was used for comparison between independent multiple groups, and SNK method was used for pairwise comparison afterwards. The categorical data were expressed as n(%), and the differences were examined by Pearson *χ*^*2*^ test or Fisher’s exact test. Spearman correlation analysis was applied, and Mann-Whitney was used to draw the estimated ROC curve. The AUC > 0.7 means that it has clinical diagnostic value, and the higher the AUC value, the higher the clinical diagnostic value. *P* < 0.05 was considered statistically significant.

## Results

### General data

In this study, there was no statistically significant difference in age and gender among Non-AECOPD group, AECOPD group and control group (*P* > 0.05) (Table 1). FEV1 and FEV1% were significantly lower in the AECOPD group than that in the Non-AECOPD group (*P* < 0.05). Besides, there was significant difference in GOLD grade between the AECOPD group and the Non-AECOPD group (*P* < 0.05) (Table 2).

**Table 1:** General data of patients

Variable	COPD group (*n* = 150)	Control group (*n* = 50)	*P* value
Age (years old)	62.23 ± 11.27	58.30 ± 11.96	0.086
Gender			0.618
Male	90 (60.00)	28 (56.00)	
Female	60 (40.00)	22 (44.00)	
BMI	22.52 ± 3.20	21.98 ± 2.72	0.381
Non-acute exacerbation			
FEV1	2.31 ± 0.56	–	
FEV1%	66.85 ± 13.51	–	
GOLD grade		–	
A	24 (16.00)		
B	109 (72.67)		
C	17 (11.33)		
Acute exacerbation			
FEV1	1.90 ± 0.56	–	
FEV1%	54.21 ± 11.52	–	
GOLD grade			
B	89 (59.33)		
C	61 (40.67)

**Table 2:** Comparison of FEV1, FEV1% and GOLD grade between AECOPD and non-AECOPD groups

Variable	AECOPD group (*n* = 150)	Non-AECOPD group (*n* = 150)	*P* value
FEV1	1.89 ± 0.56	2.31 ± 0.56	< 0.001
FEV1%	54.2 ± 11.5	66.9 ± 13.5	< 0.001
GOLD grade			< 0.001
A	0(0.00)	24 (16.00)	
B	89 (59.33)	109 (72.67)	
C	61 (40.67)	17 (11.33)	

### Hcy level in AECOPD group is significantly higher than that in non-AECOPD group

**Fig. 1:**
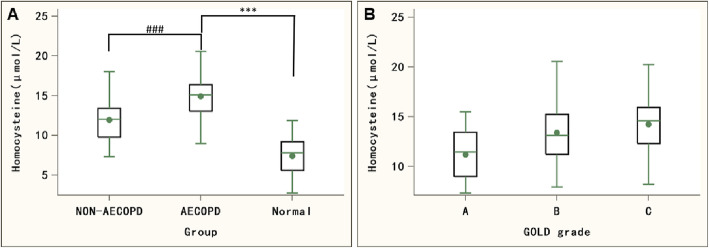
Comparsion of Hcy levels among different groups. **a** Hcy levels in subjects of Non-AECOPD group, AECOPD group and control group. ***compared with control group, P<0.001; ### compared with Non-AECOPD group, P<0.001. **b** Hcy level in patients of GOLD grade A, B and C

We found that Hcy levels in the Non-AECOPD group or the AECOPD group were significantly higher than those in the control group (*P* < 0.001). Meanwhile, compared with the Non-AECOPD group, the Hcy level in the AECOPD group was significantly higher (*P* < 0.001) (Fig. 1a). In addition, according to the classification of GOLD grade, there was significant difference in the Hcy level among different GOLD grade groups (*P* < 0.001) (Fig. 1b, Table 3).

**Table 3:** Comparison of Hcy level among different GOLD grade groups

GOLD grade	Cases	Hcy (μmol/L)	*P* value
A	24	11.49 (9.00,13.44)	< 0.001
B	198	13.14 (11.22,15.23)	
C	78	14.59 (12.31,15.96)	

### Correlation analysis between Hcy level and ventilation function

**Fig. 2:**
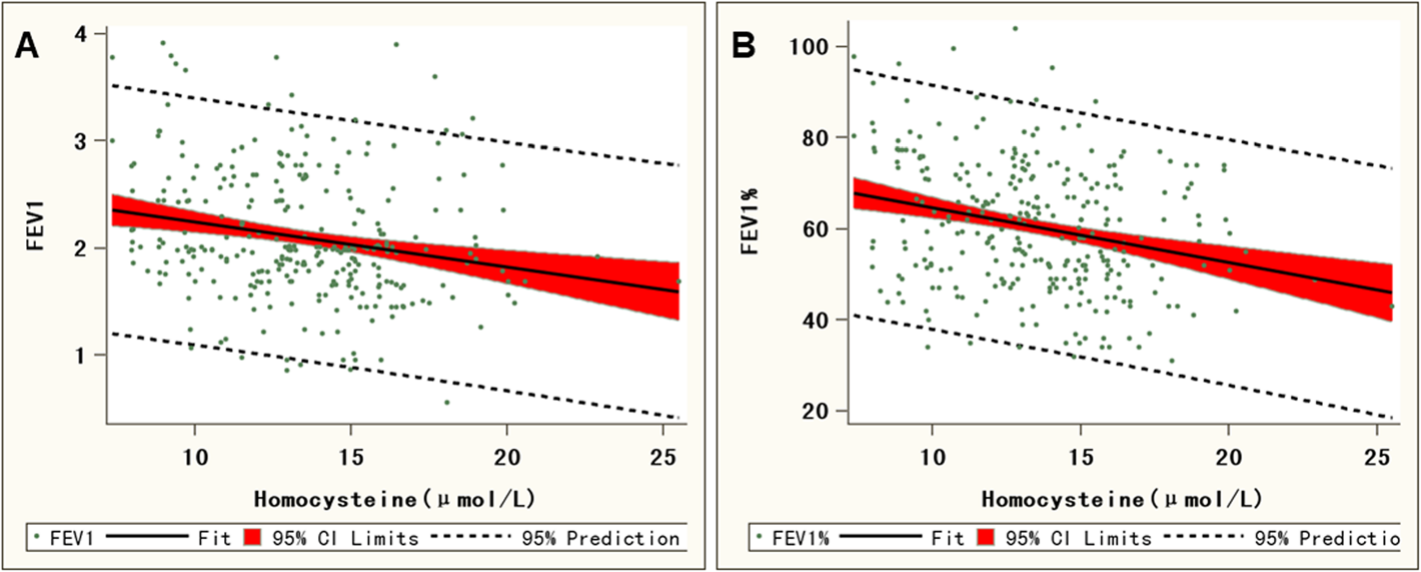
Linear regression chart of Hcy level and ventilation function index in COPD patients. **a** Linear regression chart of Hcy level and FEV1. **b** Linear regression chart of Hcy level and FEV1%

Using Spearman correlation analysis, the results showed that in the AECOPD group and the Non-AECOPD group, Hcy levels presented a negative correlation with FEV1(*r* = − 0.230). Meanwhile, FEV1% was also negatively correlated with Hcy level (*r* = − 0.256) (Fig. 2a and b, Table 4). FEV1 and FEV1% are used to determine whether the patient has ventilatory dysfunction. The lower their values, the worse the ventilatory function. Thus, these results suggested that Hcy level was positively correlated with ventilatory dysfunction.

**Table 4:** Correlation analysis of Hcy

Variable	*r* value	*P* value
FEV1	−0.230	< 0.001
FEV1%	−0.256	< 0.001

### Hcy level has predictive value for the occurrence and acute progression of COPD

**Fig. 3:**
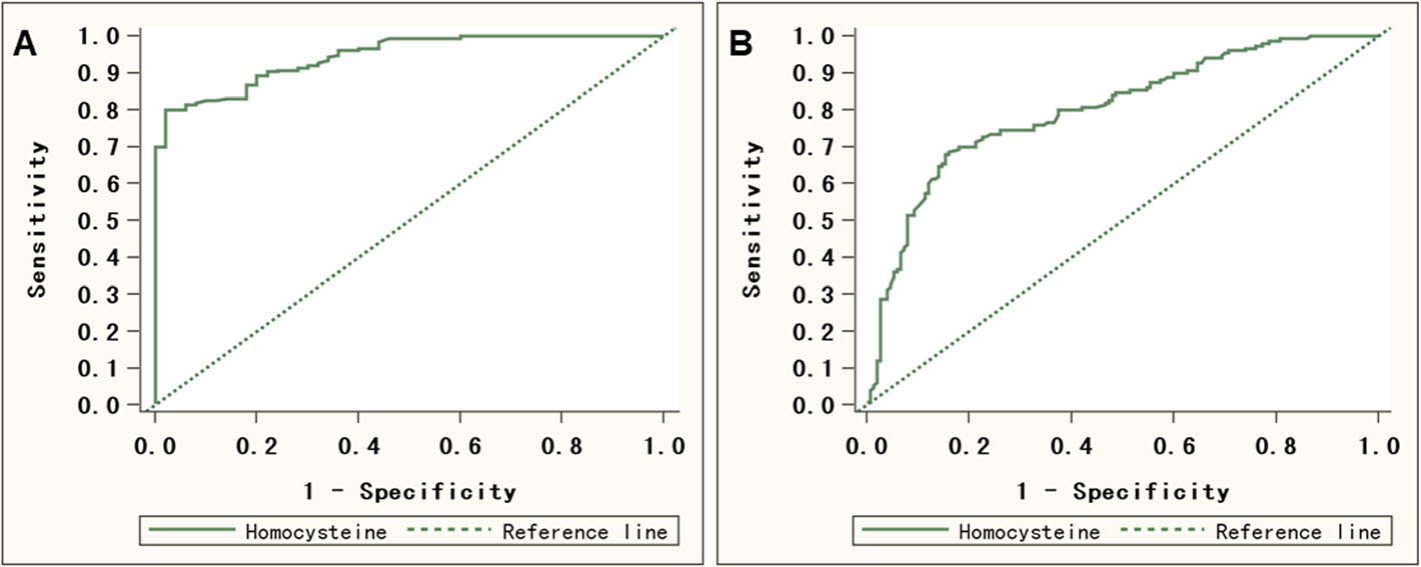
ROC curves of Hcy level. **a** ROC for identifying the occurrence of COPD. **b** ROC for identifying acute progression of COPD

ROC curve analysis was used to detect the clinical value of Hcy level for COPD, and the results showed that when the cutoff value was set to 10.8 μg/ml, the specificity, sensitivity and AUC were the best, which were 0.980, 0.800, and 0.945 (95% CI: 0.920–0.970), respectively. When Hcy was used to determine whether non-AECOPD progresses to AECOPD, our results showed that when the cutoff value was set to 14.0 μg / ml, the specificity, sensitivity and AUC were the best, which were 0.846, 0.680, and 0.802 (95% CI: 0.752–0.852), respectively. The AUC was greater than 0.7, suggesting that Hcy level has high clinical value for the occurrence and acute progression of COPD (Fig. 3a and b, Table 5).

**Table 5:** Diagnostic value of Hcy level in the prediction of occurrence and acute progression of COPD

	Occurrence	Acute progression
Sensitivity (%)	0.800	0.680
Specificity (%)	0.980	0.846
Youden’ index	0.780	0.526
AUC(95%*CI*)	0.945(0.920, 0.970)	0.802(0.752, 0.852)
Cut off value	10.8	14.0
Positive predictive value (PPV)	0.976	0.815
Negative predictive value (NPV)	0.831	0.726

In addition, compared with the prediction of acute progression of COPD, when Hcy level predicted the occurrence of COPD, its specificity (0.980 vs. 0.846, *P* < 0.001) and sensitivity (0.800 vs. 0.680, *P* < 0.001) were significantly higher, indicating that Hcy level may predict occurrence of COPD more accurately.

## Discussion

Homocysteine is an intermediate metabolite in the production of cysteine from methionine [11], metabolized through sulfur transfer pathway and trans-methylation pathway mediated by methionine synthetase, N5, N10-methylenetetrahydrofolate reductase and -cysteine synthase, which are the key enzymes of above two pathways of Hcy metabolism in vivo. Actually, genetic defects of the three metabolic enzymes and insufficient uptake of B vitamin coenzymes are the main reasons for inducing Hcy accumulation in vivo, contributing to hyperhomocysteinaemia. Fimognari et al. and Seemungal et al. have respectively demonstrated that plasma Hcy levels in COPD patients were significantly higher than those in healthy subjects, and Hcy levels were positively correlated with the severity of COPD [4, 9]. In this study, we also found the level of Hcy in AECOPD and Non-AECOPD patients were significantly higher than that in control group. The reason for the high level of Hcy in AECOPD patients may be due to the increased the consumption and insufficient intake, leading to the long-term deficiency of vitamin B in vivo.

In recent years, studies have found that Hcy, especially high levels of Hcy, is associated with lung diseases, while in the general population, Hcy is not related to the lungs [11]. In this study, we found that Hcy was positively correlated with ventilation disorders, which was consistent with previous studies [12, 13]. Studies have found that high levels of Hcy are also associated with different stages and grades of COPD. Among COPD patients, those with GOLD grades 3 and 4 have higher Hcy levels compared with those with grades 1 and 2, indicating that Hcy levels are positively correlated with GOLD grades, and high Hcy levels may be an important risk factor for the occurrence and development of disease [9]. Our study also found that there was significant difference in the Hcy level among different GOLD grade groups, and compared with patients with low-grade GOLD, those with high-grade GOLD had a higher Hcy level. These results indicated that Hcy level is closely related to the occurrence and severity of COPD,which may be an important factor in the pathogenesis of COPD.

Using ROC curve analysis, we found that the AUC was greater than 0.7, indicating that Hcy level has high clinical value for the occurrence and acute progression of COPD. The results further confirmed the close relationship between Hcy and the occurrence and progression of COPD, and also suggested the important clinical value of Hcy in determining the condition of COPD patients. However, we found that compared with the prediction of acute progression, Hcy level may predict occurrence of COPD more accurately than predict acute progression of COPD. Studies have also found that compared with hs-cTnI, hs-CRP and PCT, the diagnostic value of Hcy detection for AECOPD was lower (AUC = 0.491, 95% Cl: 0.396–0.587) [14]. In addition, Hcy is still limited as a single diagnosis basis. It has been reported that the diagnostic value of serum CRP combined with Hcy detection for acute exacerbation of COPD was better than detection of single index [15]. Hcy levels will also change as the condition improves. Some researchers conducted a pre-discharge plasma Hcy concentration test for COPD patients, and found that the Hcy concentration also showed a downward trend in patients with improved conditions [16].

The major pathogenic mechanism of Hcy is involved in mediating inflammatory substances and cytokine expression, increased number of inflammatory cells, enhanced oxidative stress response. Therefore, anti-inflammatory, improved immunity, and anti-oxidation treatments may play a therapeutic role by reducing Hcy levels. For example, using peptide drugs to supplement N-acetylcysteine can repair damaged endothelium and cells, and restore the function of alveolar epithelial cells. It has been found that COPD patients with high levels of Hcy were in a folic acid deficiency state, and folic acid supplementation can reduce Hcy levels [17]. There are also clinical data showing that the reduction of Hcy concentration can alleviate the damage of smoking to the lungs of patients, and further reduce the incidence of COPD [13]. However, the mechanism of Hcy in the pathology of COPD and the current treatment options remain limited, and more extensive and in-depth research is still needed.

There are some limitations in this study. Firstly, the sample size of this study was relatively small, and more detailed data cannot be collected due to time reasons. Secendly, our study was not able to track the condition of COPD patients for a long time and evaluate their prognosis of COPD patients. The relationship between Hcy level and the prognosis of COPD patients still needs further study. In addition, because the population of this study did not include GOLD grade D, the generalizability of this study was affected.

## Conclusion

In conclusion, Hcy level is positively correlated with the severity of COPD patients, and has predictive value for the occurrence of COPD and acute progression, thus has certain guiding significance for the diagnosis of COPD disease.

## Supplementary information

**Additional file 1.** Content and structure of the CAT questionnaire. The questionnaire include 8 questions regarding the presence of coughing, sputum, chest distress, asthma, and activity limitations caused by COPD at home and outdoors.

## Data Availability

The datasets generated and analyzed during the current study are available from the corresponding author on reasonable request.

## Abbreviations

COPD: chronic obstructive pulmonary disease
AECOPD: termed acute exacerbation COPD
VITB12: Vitamin B12
CAT: COPD assessment test
mMRC: modified UK Medical Research Council
PPV: positive predictive rate
NPV: negative predictive rate
AUC: Youden’s index and Area under the curve
SD: standard deviation
