# Supplementary material for: The diagnostic value of homocysteine for the occurrence and acute progression of chronic obstructive pulmonary disease
Source: BMC Pulm Med. 2020 Sep 7;20:237. doi: 10.1186/s12890-020-01265-w (PMC7487947; doi:10.1186/s12890-020-01265-w)
Supplement: Supplementary file 1 — Additional file 1. Content and structure of the CAT questionnaire. The questionnaire include 8 questions regarding the presence of coughing, sputum, chest distress, asthma, and activity limitations caused by COPD at home and outdoors. [file 12890_2020_1265_MOESM1_ESM.pdf]

ID : ______________________________

Gender : Male ☐ Female ☐

Age : ____________

Hospitalized Date : Date_____Month______Year________

Signature : ___________

How is your COPD?

For each item below, place a mark (√) in the box that best describes your experience.

Example: I am very happy | 0 √ 1 2 3 4 5 | I am very sad

			SCORE
I never cough	0 1 2 3 4 5	I cough all the time	☐
I have no phlegm (mucus) in my chest at all	0 1 2 3 4 5	My chest is completely full of phlegm (mucus)	☐
My chest does not feel tight at all	0 1 2 3 4 5	My chest feels very tight	☐
When I walk up a hill or one flight of stairs I am not breathless	0 1 2 3 4 5	When I walk up a hill or one flight of stairs I am very breathless	☐
I am not limited doing any activities at home	0 1 2 3 4 5	I am very limited doing activities at home	☐
I am confident leaving my home despite my lung condition	0 1 2 3 4 5	I am not at all confident leaving my home because of my lung condition	☐
I sleep soundly	0 1 2 3 4 5	I don't sleep soundly because of my lung condition	☐
I have lots of energy	0 1 2 3 4 5	I have no energy at all	☐

SCORE ☐☐
